# Patient- and family-centered performance measures focused on actionable processes of care for persistent and chronic critical illness: protocol for a systematic review

**DOI:** 10.1186/s13643-017-0476-9

**Published:** 2017-04-17

**Authors:** Louise Rose, Laura Istanboulian, Laura Allum, Lisa Burry, Craig Dale, Nicholas Hart, Claire Kydonaki, Pam Ramsay, Natalie Pattison, Bronwen Connolly, On behalf of the PERFORM study investigators, Andre Amaral, Shannon Carson, Christopher Cox, Brian Cuthbertson, Vagia Campbell, Eddy Fan, Jack Iwashyna, Vince Lo, Lorrie Hamilton, Tracey Sharon, Deepak Varma

**Affiliations:** 1 Department of Critical Care, Sunnybrook Health Sciences Centre, Toronto, ON Canada; 2 Lawrence S. Bloomberg Faculty of Nursing and Interdepartmental Division of Critical Care Medicine, University of Toronto, 155 College St. Suite 276, Toronto, ON Canada; 3 Provincial Centre of Weaning Excellence, Michael Garron Hospital, 825 Coxwell Ave, East York, ON M4C 3E7 Canada; 4 Lane Fox Respiratory Unit, St Thomas’s Hospital, Guy’s and St Thomas’s NHS Foundation Trust, Westminster Bridge Rd, London, UK; 5 Leslie Dan Faculty of Pharmacy, Mount Sinai Hospital, University of Toronto, 600 University Ave, Rm 18-377, Toronto, ON Canada; 6 Lawrence S. Bloomberg Faculty of Nursing, University of Toronto, 155 College St. Rm 286, Toronto, ON Canada; 7 Respiratory and Critical Care Medicine Department of Asthma, Allergy, and Respiratory Science Division of Asthma, Allergy and Lung Biology, King’s College London, London, UK; 8 Adult Nursing, School of Health & Social Care, Teaching Fellow of the Academy of Higher Education, Edinburgh Napier University, Sighthill Campus, Sighthill Court, rm 3.b46, Edinburgh, Scotland EH11 4BN; 9 Nursing Studies, Edinburgh Napier University, Sighthill Campus, Sighthill Court, room rm 3B.45, Edinburgh, Scotland EH11 4BN; 10 The Royal Marsden NHS Foundation Trust, Dovehouse DB3, Fulham Rd, London, SW36J UK; 11 Lane Fox Clinical Respiratory Physiology Research Centre, Westminster Bridge Rd, London, UK; 12 Guy’s and St. Thomas’ NHS Foundation and King’s College London NIHR Biomedical Research Centre, London, UK; 13 Centre for Human and Aerospace Physiological Sciences, King’s College London, London, UK

**Keywords:** Chronic critical illness, Persistent critical illness, Quality indicator, Process of care, Intensive care

## Abstract

**Background:**

Approximately 5 to 10% of critically ill patients transition from acute critical illness to a state of persistent and in some cases chronic critical illness. These patients have unique and complex needs that require a change in the clinical management plan and overall goals of care to a focus on rehabilitation, symptom relief, discharge planning, and in some cases, end-of-life care. However, existing indicators and measures of care quality, and tools such as checklists, that foster implementation of best practices, may not be sufficiently inclusive in terms of actionable processes of care relevant to these patients. Therefore, the aim of this systematic review is to identify the processes of care, performance measures, quality indicators, and outcomes including reports of patient/family experience described in the current evidence base relevant to patients with persistent or chronic critical illness and their family members.

**Methods:**

Two authors will independently search from inception to November 2016: MEDLINE, Embase, CINAHL, Web of Science, the Cochrane Library, PROSPERO, the Joanna Briggs Institute and the International Clinical Trials Registry Platform. We will include all study designs except case series/reports of <10 patients describing their study population (aged 18 years and older) using terms such as persistent critical illness, chronic critical illness, and prolonged mechanical ventilation. Two authors will independently perform data extraction and complete risk of bias assessment. Our primary outcome is to determine actionable processes of care and interventions deemed relevant to patients experiencing persistent or chronic critical illness and their family members. Secondary outcomes include (1) performance measures and quality indicators considered relevant to our population of interest and (2) themes related to patient and family experience.

**Discussion:**

We will use our systematic review findings, with data from patient, family member and clinician interviews, and a subsequent consensus building process to inform the development of quality metrics and tools to measure processes of care, outcomes and experience for patients experiencing persistent or chronic critical illness and their family members.

**Systematic review registration:**

PROSPERO CRD42016052715

**Electronic supplementary material:**

The online version of this article (doi:10.1186/s13643-017-0476-9) contains supplementary material, which is available to authorized users.

## Background

Advances in technology, establishment of evidence for efficacy of interventions, and subsequent adoption into practice have improved intensive care unit (ICU) survival rates [1]. Notwithstanding these advancements, approximately 10% of critically ill patients transition from acute critical illness to a state of persistent and in some cases chronic critical illness [2]. Persistent critical illness is reflected by ongoing critical illness and some degree of instability that is no longer directly attributable to the original reason for ICU admission [3]. Patients with chronic critical illness, in contrast, usually experience relative clinical stability but continue to require prolonged ICU stays and, in most cases, a prolonged need for mechanical ventilation [4–6]. Patients with persistent or chronic critical illness (P/CCI) experience a syndrome of ongoing organ dysfunction, profound weakness, extreme symptom burden, and ongoing physical and cognitive deficits resulting in a difficult and prolonged course of recovery [5, 7]. Family members also experience significant physical and psychological burden, including anxiety, depression, and post-traumatic stress disorder that arguably require an enhanced level of support [8].

A recent population-based study of over one million ICU patients in Australia and New Zealand identified 5% experienced persistent critical illness defined as a length of stay of ≥10 days, but accounted for 33% of ICU bed days and 15% of hospital bed days [9]. Similar numbers in terms of persistent or chronic critical illness have been reported in the UK [10], Canada [11], and the USA [2]. High healthcare utilization results in increased healthcare costs with one US study estimating the attributable costs as high as $35 billion or 1.4% of the US healthcare expenditure [12]. Therefore, these patients contribute small actual numbers but high cost and care delivery burden to the ICU, hospital, and healthcare system. Moreover, these patients experience worse clinical outcomes with hospital mortality (20 to 49% [13]) and 1-year mortality (32 to 55% [14]) substantially higher than patients that require a short ICU admission. Less than half of these patients that survive ICU admission will return home and therefore require post-acute hospitalization institutional care [9].

Patients with P/CCI have unique and complex needs that may be distinct from those of patients at the acute stage of ICU admission and medical stabilization. Therefore, these patients require a change in the clinical management plan and overall goals of care to a focus on rehabilitation, symptom relief, discharge planning, and in some cases, ventilation discontinuation or end-of-life care [15]. Despite increasing recognition of the burden of P/CCI for patients, families, and the healthcare system, and differences in their care needs [16], few studies have been conducted to inform development of ICU care quality measures focused on actionable processes of care specific to this patient population. Processes comprise things that we do, or fail to do for our patients, i.e., treatment and care [17]. Actionable processes of care are those that clinicians have direct control over [18], i.e., commencing the weaning process or discontinuing sedation. Our group has identified that processes of care such as weaning and mobilization protocols *infrequently* include guidance specific to patients with P/CCI [19]. Moreover, few ICUs incorporate into practice those assessments and interventions deemed pertinent including communication adjuncts, anxiety and dyspnea assessment, and access to psychiatric services during ICU admission [19]. Quality indicators are tools that *indicate* care quality and compare actual care against ideal criteria [20]. Performance measures are tools to *measure* care quality. Existing indicators of ICU care quality such as rates of central line infection, ventilator-associated pneumonia, and unplanned extubation [21], and tools such as checklists that measure implementation of best practice [22], may not be sufficiently inclusive in terms of actionable processes of care relevant to patients with P/CCI.

At present, it is unclear if the absence of performance measures for potentially actionable processes of care specifically designed for patients with P/CCI contribute to poor experience and adverse outcomes. Provision of optimal care for these patients has implications for health care spending on critical care services [10], health care quality outcomes, and timely integration of appropriate services including chronic illness management, rehabilitation, community services, and palliative care. Additionally, the number of patients experiencing P/CCI is predicted to grow exponentially over coming years due to population aging and increased morbidity and thus the need to optimize care for these patients is imperative [12]. Therefore, our objective is to identify the processes of care, performance measures, quality indicators, and outcomes including reports of patient/family experience described in the current evidence base that are relevant to P/CCI patients and their family members.

## Methods

We prepared this review protocol using the Preferred Reporting Items for Systematic Review and Meta-Analyses Protocol (PRISMA-P) guidelines [23] and completed the PRISMA-P checklist (Additional file 1). We registered the protocol on International Prospective Register of Systematic Reviews (PROSPERO) CRD42016052715.

### Data sources and search strategy

We created a preliminary search strategy (Additional file 2) as an iterative process with guidance from an experienced information specialist. Search strategies will utilize a combination of controlled vocabulary (e.g., “Intensive Care Units”, “Critical illness”) and keyword combinations (e.g., chronic or persistent) adjacent to (acute or critical or intensive). Vocabulary and syntax will be adjusted across databases. We will remove animal-only studies and opinion pieces (e.g., editorials, letters). As we are including a range of study designs, we will not apply study-specific filters.

Prior to search execution, a second information specialist used the Peer Review for Electronic Search Strategies (PRESS) template [24, 25] to review and approve the search strategy. We will search the following electronic databases (March 1980 to Nov 2016): Medline, CINAHL, Embase, and the Web of Science. We will search systematic review databases including the Cochrane Library, PROSPERO, and the Joanna Briggs Institute. We will search major guideline sites (e.g., CMA Infobase, National Guideline Clearinghouse) for clinical practice guidelines and policy documents, and websites of relevant professional societies for practice recommendations relevant to our population of interest. We will examine reference lists of relevant studies/reviews for additional studies and will contact corresponding authors for details of additional published/unpublished work. We will search for unpublished and ongoing trials on the http://apps.who.int/trialsearch website.

### Study selection—inclusion and exclusion criteria

We will include all study designs except case series/reports of less than 10 patients. We will exclude narrative and systematic reviews but will screen those relevant to our population of interest for discussion of additional processes of care, quality indicators, or outcomes not present in primary studies. We will exclude commentaries, editorials, and opinion papers, and for pragmatic reasons, studies reported in languages other than English.

### Population

We will include studies describing their study population (aged 18 years and older) using terms such as persistent critical illness, chronic critical illness, and prolonged mechanical ventilation or that describe a study population admitted to a specialized weaning facility, long-term acute care hospital, or respiratory high dependency unit. Based on previous experience with this literature [26], we are aware such terms are highly variable in the duration of mechanical ventilation or ICU length of stay used to define the study cohort. Therefore, we will include only studies that report on a cohort with an a priori ICU length of stay of 7 days or more as an inclusion criterion for the study (not as the mean or median duration of stay of the enrolled cohort for that study). We have selected this definition based on the consensus definition used by Medicare and Medicaid in the USA [27]. We will exclude studies describing a long-term mechanical ventilation population, defined as patients with no expectation of weaning, from care venues other than those described above, and studies describing patient cohorts receiving mechanical ventilation at home.

### Intervention

We will include studies reporting on actionable (i.e., those that clinicians have direct control over) processes of care (e.g., weaning, mobilization, or sleep protocols; symptom assessment; family involvement) or interventions (e.g., communication devices) specific to our patient population of interest. We will include studies reporting the experiences of patients with P/CCI and their family members, clinician perspectives, as well as studies describing unit organizational structure (e.g., staffing, daily routines) relevant to these patients. We will exclude studies examining predictors of P/CCI and those describing only cohort characteristics and outcomes (e.g., length of stay, mortality) without description of processes of care, intervention, or patient or family experience.

### Comparator

We will include studies that compare processes of care or interventions to usual care, another process of care or intervention, as well as studies without a comparator group.

### Outcomes

Our primary outcome is to determine actionable processes of care and interventions deemed relevant to P/CCI patients and their family members. Secondary outcomes include (1) quality indicators (tools that indicate care quality) and performance measures (measures of quality) considered relevant to our population of interest and (2) themes related to patient and family experience.

### Study selection and data extraction

Using a pre-designed screening tool listing inclusion and exclusion criteria, two authors (LR/LI) will independently examine study titles and abstracts. Screening will be managed using Endnote 17; all decisions will be recorded in an Excel file. We will obtain full text articles considered potentially relevant by either author and examine for eligibility. Disagreements will be resolved through discussion and referred to a third author for arbitration if necessary. Two authors in pairs will independently extract study data using a standardized form; all data extraction will be checked for accuracy by a third author. We will extract data on the country(ies) study was performed in, care venue type and characteristics, patient age range, diagnostic categories, severity of illness, inclusion and exclusion criteria, verbatim description of care processes, interventions, and performance measures as well as themes related to patient and family experience. We will also extract study outcomes, i.e., what was measured, how it was measured, and study results. Data extractors will not be blinded to study citations.

### Risk of bias reporting

Two investigators will independently assess risk of bias using the Cochrane Risk of Bias tool for randomized and quasi-randomized studies and the Scottish Intercollegiate Guidelines Network (SIGN) checklists for cohort and case–control studies for non-randomized studies [28]. For qualitative studies, we will assess study quality using the multi-dimensional concept of quality recommended by the Cochrane Quality and Intervention Methods Group [29] that includes (1) quality of reporting, e.g., explicitness in reporting all study aspects; (2) methodological rigor, e.g., validity and reliability of study design and process; and (3) overall conceptual depth and breadth, e.g., if stated study aims, rationale, or theory (if the study is explicitly theoretically informed) are reflected in the study design, process, and findings. We will also determine if the methods and conceptual underpinning are congruent. Two authors will independently appraise study quality using the 10 questions of the 2014 Critical Appraisal Skills Programme (CASP) quality assessment tool for qualitative studies [30]. Each of these 10 questions will be answered as “yes”, “no”, or “unclear”. The CASP tool does not consider the more conceptual or theoretical aspects of qualitative studies, the same two authors will independently appraise study quality using the seven criteria outlined by Popay [31]. These seven criteria include (1) Illumination of subjective meaning; (2) Adaptation and responsiveness of research design; (3) Sample provides appropriate knowledge; (4) Description sufficiently detailed; (5) Sources of knowledge compared and contrasted; (6) Shift from description to analysis and interpretation; and (7) Claims for generalizability. We will present quality assessments in tabular format.

### Approach to evidence synthesis, analysis, and interpretation

We will summarize our search results in a PRISMA study flow diagram [32]. We will generate summary tables reporting counts and proportions (categorical variables) and means and standard deviations or median and interquartile ranges (continuous variables) as reported by study authors of study and cohort characteristics as well as study results. We will catalogue descriptions of care processes, interventions, quality metrics, and tools described in eligible studies. Due to the anticipated diversity in study designs as well as diversity in processes of care, interventions, quality indicators, and measures under evaluation of included studies, we will not perform meta-analyses but will generate a narrative synthesis of study results. We anticipate qualitative data will describe patient and family experience or clinician perspectives. We will generate tables of author reported categories, themes, and subthemes. We will undertake content analysis [33, 34] to quantify common categories and themes within these categories. We will categorize quality of care components from quantitative and qualitative studies using the conceptual framework of structure, process, and outcomes [35]. We will generate descriptive statistics using SPSS Version 24 (Armonk, NY).

### Dealing with missing data

We (LR) will attempt to contact study authors for unreported data or clarification of study methods using a maximum of three e-mails. If data remains unavailable, we will analyze the available data and report the potential impact of missing data in the discussion section.

### Subgroup and sensitivity analyses

We do not plan to undertake subgroup or sensitivity analyses.

### Publication bias

We do not intend to examine publication bias.

## Discussion

The existence of a distinct cohort of relatively low volume, high cost, and poor outcome patients requiring mechanical ventilation and ICU admission for longer than the average patient has long been recognized [2, 9, 10, 36–38]. However, for the most part, studies of the effectiveness of interventions in the ICU focus on the acute phase of critical illness. Similarly, quality indicators, measures, and tools to evaluate quality of care, and patient or family member experience, have not been developed with patients with P/CCI in mind, nor have they been developed with the patient and family perspective at the forefront. At present, the absence of quality and performance measures for potentially actionable processes of care specifically designed for patients with P/CCI may contribute to poor experience and adverse outcomes.

This review is the first step in an item generation process to identify processes of care, quality indicators, performance measures, and outcomes that are important from the perspective of patients experiencing P/CCI, their family members, and the clinicians that treat them. Subsequent phases of this work include further item generation via ICU survivor, family member, and clinician interviews. This phase will be followed by item reduction via rigorous consensus based methods including a two-round Delphi process and an adaptation of the nominal group technique used for priority setting partnerships by the James Lind Alliance. The James Lind Alliance is a non-profit organization that pioneered setting priorities for “treatment uncertainties” of direct relevance and benefit to patients and clinicians using the principles of equal contribution and transparency [39]. Our overall aim is to inform the development of tools to improve care quality and experience of patients experiencing P/CCI and their family members.

## Conclusion

In summary, patients with P/CCI are a small but high cost and care delivery burden that experience poor clinical outcomes. Due to their unique and complex needs, existing indicators and measures of ICU care quality and performance may not be sufficiently relevant to these patients. Our systematic review comprises the first phase of the development of quality metrics and tools designed to measure processes of care, outcomes, and experience relevant of this patient population, their family members, and the clinicians that treat them.

### Registration

This systematic review is registered with PROSPERO, an international prospective register of systematic reviews: https://www.crd.york.ac.uk/PROSPERO/display_record.asp?ID=CRD42016052715.

## Additional files

Additional file 1: PRISMA-P 2015 Checklist. (DOCX 35 kb)

Additional file 2: MEDLINE search strategy. (DOCX 15 kb)

## Abbreviations

CASP: Critical Appraisal Skills Programme
ICU: Intensive care unit
P/CCI: Persistent or chronic critical illness
PRESS: Peer Review for Electronic Search Strategies
PRISMA: Preferred Reporting Items for Systematic Reviews and Meta-Analysis
