# Supplementary material for: Patient- and family-centered performance measures focused on actionable processes of care for persistent and chronic critical illness: protocol for a systematic review
Source: Syst Rev. 2017 Apr 17;6:84. doi: 10.1186/s13643-017-0476-9 (PMC5392946; doi:10.1186/s13643-017-0476-9)
Supplement: Supplementary file 2 — MEDLINE search strategy. (DOCX 15 kb) [file 13643_2017_476_MOESM2_ESM.docx]

Additional File 2

MEDLINE search strategy

1 Intensive Care Units

2 Burn Units

3 Coronary Care Units

4 Respiratory Care Units

5 exp Critical Care

6 ((intensive or critical or acute) adj3 care).tw,kw.

7 (ICU or ICUs or SICU or SICUs or CCU or CCUs).tw,kw.

8 (burn? adj3 (unit? or centre? or center?)).tw,kw.

9 ((cardiac or coronary or heart) adj3 (unit? or centre? or center?)).tw,kw.

10 (respiratory adj3 (unit? or centre? or center?)).tw,kw.

11 ((surgical or surger*) adj3 (unit? or centre? or center?)).tw,kw.

12 (high dependency adj3 (unit? or centre? or center?)).tw,kw.

13 ((stepdown or step-down) adj3 (unit? or centre? or center?)).tw,kw.

14 (HDU or HDUs or SDU or SDUs or EDSDU or EDSDUs).tw,kw.

15 ((special* or dedicated or intens*) adj weaning adj3 (unit? or centre? or center? or program* or ward?)).tw,kw.

16 or/1-15 [INTENSIVE CARE]

17 Chronic Disease/ and Critical Illness/

18 (((chronic* or persist*) adj (acute* or critical* or intens*)) and (ill or illness* or sick or sickness* or care)).tw,kw.

19 ((long term or longterm or prolong* or protract*) adj2 acute care).tw,kw.

20 (LTAC or LTACH).tw,kw.

21 ((extend* or extensive or lengthy or long or long-term or longterm or prolong* or protract*) adj2 (stay or stays or "length of stay*" or "lengths of stay*" or LOS)).tw,kw.

22 ((difficult* or extend* or extensive or long or long-term or longterm or prolong* or protract*) adj2 (ventilat* or wean*)).tw,kw.

23 ((long-term or longterm or prolong* or protract*) adj2 intensive care).tw,kw.

24 ((long-term or longterm or prolong* or protract*) adj2 critical care).tw,kw.

25 ((extend* or extensive or long-term or longterm or prolong* or protract*) adj2 (ICU or ICUs or SICU or SICUs or CCU or CCUs)).tw,kw. (

26 ((extend* or extensive or long-term or longterm or prolong* or protract*) adj2 (HDU or HDUs or SDU or SDUs or EDSDU or EDSDUs)).tw,kw.

27 ((more than 7 days* or greater than 7 days* or beyond 7 days*) adj2 (stay or stays or "length of stay*" or "lengths of stay*" or LOS)).tw,kw.

28 ((more than seven days* or greater than seven days* or beyond seven days*) adj2 (stay or stays or "length of stay*" or "lengths of stay*" or LOS)).tw,kw.

29 ((8 days* or 9 days* or 10 days* or 11 days* or 12 days* or 13 days* or 14 days* or 15 days* or 16 days* or 17 days* or 18 days* or 19 days* or 20 days* or 21 days*) adj2 (ventilat* or wean*)).tw,kw.

30 ((eight days* or nine days* or ten days* or eleven days* or twelve days* or thirteen days* or fourteen days* or fifteen days* or sixteen days* or seventeen days* or eighteen days* or nineteen days* or twenty days* or twenty-one days*) adj2 (ventilat* or wean*)).tw,kw.

31 ((1 week or one week or 2 weeks* or two weeks* or 3 weeks* or three weeks*) adj2 (ventilat* or wean*)).tw,kw.

32 or/17-31 [CHRONIC CRITICAL ILLNESS/CARE/WEANING/VENTILATION]

33 16 and 32 [INTENSIVE CARE & CHRONIC CRITICAL ILLNESS/CARE/WEANING/VENTILATION]

34 exp Child/ not (exp Child/ and exp Adult/)

35 exp Infant/ not (exp Infant/ and exp Adult/)

36 33 not (34 or 35) [CHILD-ONLY REMOVED]

37 exp Animals/ not (exp Animals/ and Humans/)

38 36 not 37 [ANIIMAL-ONLY REMOVED]

39 (comment or editorial or interview or news or newspaper article).pt.

40 (letter not (letter and randomized controlled trial)).pt.

41 38 not (39 or 40) [OPINION PIECES REMOVED]

42 limit 41 to yr="1980-current"

43 limit 42 to english language
